# Relations between plasma microRNAs, echocardiographic markers of atrial remodeling, and atrial fibrillation: Data from the Framingham Offspring study

**DOI:** 10.1371/journal.pone.0236960

**Published:** 2020-08-19

**Authors:** Aditya Vaze, Khanh-Van Tran, Kahraman Tanriverdi, Mayank Sardana, Darleen Lessard, J. Kevin Donahue, Bruce Barton, Gerard Aurigemma, Steven A. Lubitz, Honghuang Lin, George H. Nasr, Amiya Mandapati, Emelia J. Benjamin, Ramachandran S. Vasan, Jane E. Freedman, David D. McManus

**Affiliations:** 1 Division of Cardiology, Department of Medicine, University of Massachusetts Medical School, Worcester, Massachusetts, United States of America; 2 Division of Epidemiology of Chronic Diseases, Department of Quantitative Health Sciences, University of Massachusetts Medical School, Worcester, Massachusetts, United States of America; 3 Biostatistics and Health Services Research, Department of Quantitative Health Sciences, University of Massachusetts Medical School, Worcester, Massachusetts, United States of America; 4 Cardiovascular Research Center, Massachusetts General Hospital, Boston, Massachusetts, United States of America; 5 National Heart Lung and Blood Institute's and Boston University's Framingham Heart Study, Framingham, Massachusetts; Computational Biomedicine Section, Department of Medicine, Boston University School of Medicine, Boston, Massachusetts, United States of America; 6 Department of Medicine, University of California Irvine, Orange, California, United States of America; 7 Department of Medicine, and Department of Epidemiology, Boston University's and National Heart, Lung, and Blood Institute's Framingham Heart Study, Framingham, Massachusetts; Section of Preventive Medicine and Epidemiology and Cardiovascular Medicine, Boston University Schools of Medicine and Public Health, Boston, Massachusetts, United States of America; Scuola Superiore Sant'Anna, ITALY

## Abstract

**Background:**

Circulating microRNAs may reflect or influence pathological cardiac remodeling and contribute to atrial fibrillation (AF).

**Objective:**

The purpose of this study was to identify candidate plasma microRNAs that are associated with echocardiographic phenotypes of atrial remodeling, and incident and prevalent AF in a community-based cohort.

**Methods:**

We analyzed left atrial function index (LAFI) of 1788 Framingham Offspring 8 participants. We quantified expression of 339 plasma microRNAs. We examined associations between microRNA levels with LAFI and prevalent and incident AF. We constructed pathway analysis of microRNAs’ predicted gene targets to identify molecular processes involved in adverse atrial remodeling in AF.

**Results:**

The mean age of the participants was 66 ± 9 years, and 54% were women. Five percent of participants had prevalent AF at the initial examination and 9% (n = 157) developed AF over a median 8.6 years of follow-up (IQR 8.1–9.2 years). Plasma microRNAs were associated with LAFI (N = 73, p<0.0001). Six of these plasma microRNAs were significantly associated with incident AF, including 4 also associated with prevalent AF (microRNAs 106b, 26a-5p, 484, 20a-5p). These microRNAs are predicted to regulate genes involved in cardiac hypertrophy, inflammation, and myocardial fibrosis.

**Conclusions:**

Circulating microRNAs 106b, 26a-5p, 484, 20a-5p are associated with atrial remodeling and AF.

## Introduction

Atrial fibrillation (AF) is the world’s most common arrhythmia, affecting over 46 million individuals worldwide in 2016, and the prevalence of AF is increasing [1]. Although AF is strongly related to the duration and the intensity of exposure to traditional cardiovascular risk factors, a substantial portion of an individual’s risk for AF is unexplained by known AF risk factors [2,3]. Although AF is heritable, allelic variation does not fully account for AF heritability, suggesting that other genetic or epigenetic factors may contribute to the development of a substrate vulnerable to this arrhythmia [4].

Micro-ribonucleic acids (microRNAs) are short, endogenous non-coding RNAs that regulate post-transcriptional gene expression that are integral to cardiac structure and function [4,5]. In addition to their direct physiologic and pathologic roles, microRNAs are readily detectable in the circulation and may provide insights into gene expression in tissues in several acute and chronic cardiovascular diseases (CVD), including acute myocardial infarction, heart failure, and AF [6,7]. MicroRNAs also have provided some insight into gene regulatory networks implicated in the pathogenesis of CVD and may represent attractive therapeutic targets.

Atrial structural remodeling, as measured echocardiographically by left atrial (LA) size and LA phasic function, is a potent intermediate phenotype that reflects prior CVD risk factor exposure intensity and has been associated with risk for development of AF [8]. We recently demonstrated strong associations between echocardiographic LA function index (LAFI), a composite measure of both LA structure and function, with incident AF and CVD in the Framingham Offspring Cohort [9]. LAFI, unlike other echocardiographic parameters, captures AF vulnerability even in the presence of normal LA size because it incorporates atrial function in addition to volume-based measurements.

Experimental studies have shown that exposure to cardiovascular risk factors influences LA gene expression, pathological cardiac remodeling, and AF [10]. Recently, we demonstrated that plasma microRNAs related strongly to AF as well as recurrence of AF after catheter ablation [11,12]. In the present study, we employed a mechanism-based framework to identify promising candidate plasma microRNAs and then explored associations of those microRNAs with incident and prevalent AF in a community-based cohort.

## Materials and methods

### Study population

The data, analytic methods, and study materials have been made available to other researchers for purposes of reproducing the results or replicating the procedure. The data have been deposited in dbGaP (https://www.ncbi.nlm.nih.gov/gap) under the accession number phs000007 [13].

**Fig 1:**
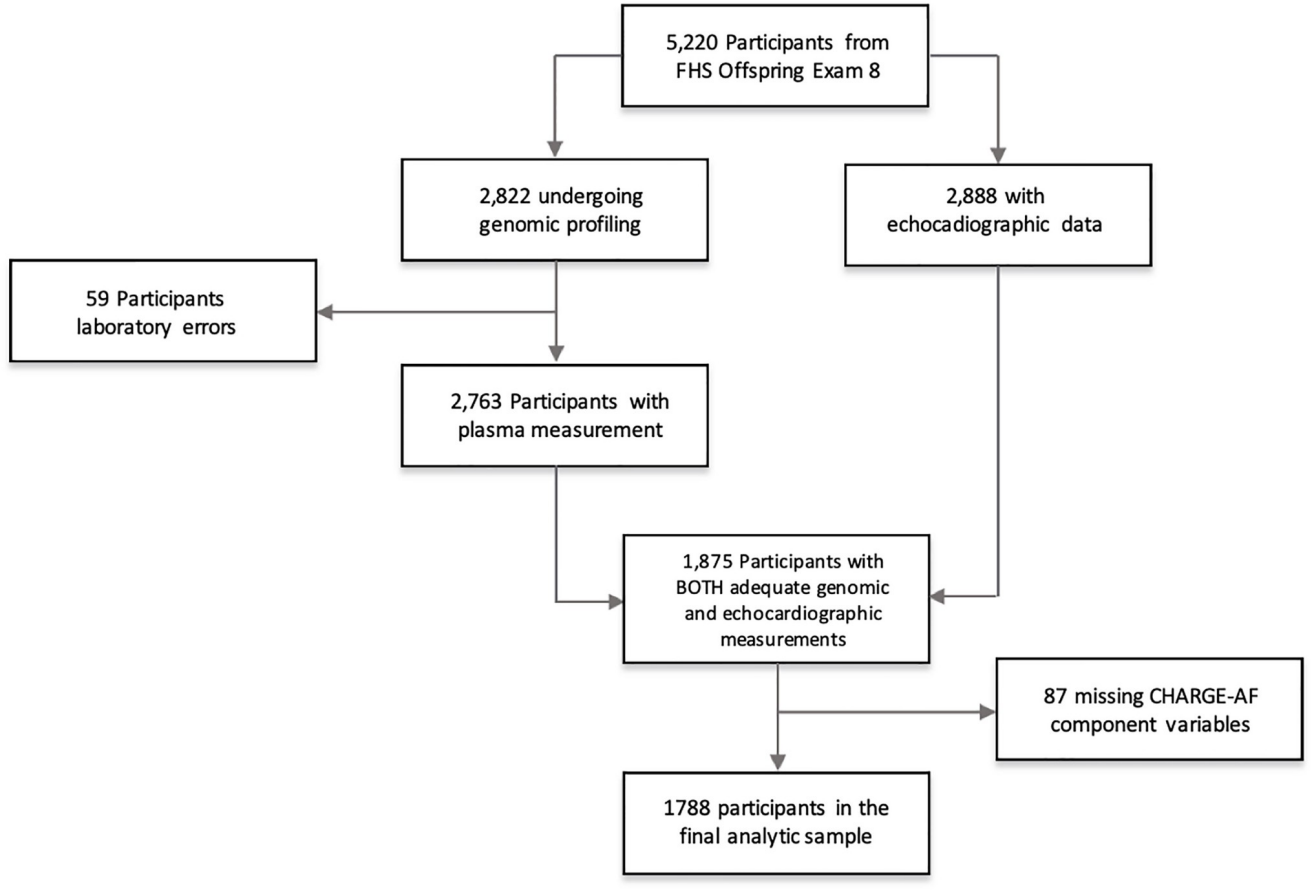
Enrollment, screening, and transcriptomic profiling and echocardiographic measurements of the framingham offspring exam 8 cohort.

The Framingham Offspring Study is an ongoing longitudinal cohort study that started in 1971 with the enrollment of the children of the Original Framingham Heart Study cohort [14]. The participants are serially evaluated every 4 to 8 years. A total of 2888 Offspring Study participants underwent 2-dimensional transthoracic echocardiogram with digital image acquisition during examination 8 (2005–2008). For the present analysis, we excluded the participants with suboptimal LA imaging, and those with incomplete data for key covariates, including components of the Cohorts for Heart and Aging Research in Genomic Epidemiology–Atrial Fibrillation (CHARGE-AF) risk score (Fig 1) [15]. The baseline characteristics of FHS Offspring participants with and without measurable LAFI did not differ [9]. The protocol for the Framingham Offspring study was approved by the Boston University Medical Center Institutional Review Board and all analyses were approved by the University of Massachusetts Medical School Institutional Review Board (IRB #H0010802). All participants provided written informed consent.

### Ascertainment of AF

During each examination cycle a study physician obtains medical history and performs a detailed physical examination on each participant. Participants are asked whether they have been diagnosed with AF. A 12-lead electrocardiogram is obtained during each examination. Medical records spanning interim hospitalizations and clinic visits are reviewed by study physicians and potential incident AF cases are adjudicated by two cardiologists. AF is confirmed if the arrhythmia is seen on a 12-lead electrocardiogram, telemetry recording, or Holter monitor tracing by trained cardiologist over-readers (DDM, SAL, EJB). Any AF diagnosed at or before examination 8 was considered prevalent AF, whereas AF newly-diagnosed at any point through December 31, 2014 during the follow-up period after examination 8 was considered incident AF.

### MicroRNA profiling and selection

As part of a genomic/transcriptomic profiling study, a total of 1875 Framingham Offspring Study participants underwent venipuncture for whole blood collection during examination cycle 8. Plasma was isolated from blood samples. The methods for processing blood samples, storing plasma samples, RNA isolation, and microRNA profiling are discussed in S1 Text *Supplement*: *Methodology* and have also previously been described [16]. The microRNA profiling of plasma was performed at the high-throughput Gene Expression Core Laboratory at the University of Massachusetts Medical School.

### Echocardiographic measurements

The Framingham Offspring Study employs a standardized protocol for 2-dimensional and Doppler echocardiographic imaging using parasternal long- and short-axis views in addition to the apical views [17]. In brief, Simpson’s biplane summation of disks method was used to make LA volume measurements in apical 2-chamber and 4-chamber views [17]. Maximum and minimum LA volumes (LAmax, LAmin) were calculated by averaging the respective volumes in apical 2- and 4-chamber views. LAmax was indexed to the body surface area to calculate LAmax index. Stroke volume was calculated as the difference of left ventricular end-diastolic and end-systolic volumes. Left ventricular outflow tract (LVOT) diameter was measured in the parasternal long axis view. LVOT velocity-time integral (LVOT-VTI) was derived by dividing the stroke volume by LVOT area $\left(3.14\times (\frac{LVOT\;diameter}{2})\right)$ [18]. Offline measurements of LA volumes were performed using the Digisonics DigiView System Software (version 3.7.9.3, Digisonics, Inc, Houston, Texas, USA). LA emptying fraction was calculated as $(\frac{LA\text{max}-\;LA\;min}{LA\;max})\times \;100$. A previously validated formula was used to calculate LAFI [19]. It is $\left(\frac{LA\;emptying\;fraction\;\times \;LVOT\;-VTI}{LA\text{max}index}\right)$.

### Statistical analyses

Descriptive statistics were noted with means and standard deviations for continuous variables and with counts and percentages for categorical variables. A statistically robust, two-step analysis model was used to leverage a quantitative, intermediate phenotype to identify candidate microRNAs and inform a mechanism-based, hypothesis-driven framework for examining microRNA-AF associations. In step 1, we examined the relations between plasma microRNAs with LAFI. In step 2, we examined the associations of microRNAs identified from step 1 with prevalent and incident AF.

For step 1 of our analyses, we used ordinary least-squares linear regression to quantify associations between microRNA levels and LAFI in all participants with AF. We adjusted for components of the augmented CHARGE-AF model, a composite risk score based on various clinical and electrocardiographic risk factors, including: age, race, height, weight, systolic blood pressure, diastolic blood pressure, current smoking, antihypertensive medication use, diabetes, prior myocardial infarction, heart failure, and electrocardiographic PR interval and left ventricular hypertrophy [15]. This risk score was selected because it was developed for prediction of AF [20,21]. To account for multiple testing, we employed Bonferroni correction to establish a more restrictive threshold for defining statistical significance. The α for achieving significance was set at 0.05/340 = 0.000147 *a priori*. We then compared C_q_ with LAFI directly. Note that C_q_ represents an inverse log measure of concentration, with exponentiation factor 2. C_q_ is not normally distributed and microRNA concentrations are reported as medians and IQR.

In step 2 of the analysis, we examined the associations of microRNAs identified from step 1, with prevalent AF using a logistic regression model, and incident AF with a Cox regression model. Here, the continuous C_q_ values, which correspond to the inverse of plasma microRNA levels, were compared with AF status. Lastly, we calculated Kaplan-Meier estimators to compare differences in time to incident AF as a function of plasma microRNA levels. For this, we used 1/C_q_ as a surrogate for plasma levels and then dichotomized our continuous data based on median value of 1/C_q_. In our graphical representation of the Kaplan-Meier curve, we chose to use 1/C_q_, as it directly corresponds to microRNA plasma levels, and thus best conveys our message regarding relationships between plasma levels and AF status. Proportional hazards (PH) assumptions were checked and none of the models of miRs that are related to AF violated the PH assumptions.

To avoid over-fitting our statistical model, we did not re-perform adjustments for components of the augmented CHARGE-AF in step 2, as these adjustments had already been made in step 1. Furthermore, we did not perform a second correction for multiple testing when LAFI-associated microRNAs were examined in relation to AF. This decision was defined *a priori* as multiple steps of correction was deemed overly conservative and inconsistent with methods employed in similar studies [22–24].

Note that a table of the distribution of correlations for all microRNAs (S1 Table), a pairwise correlation matrix (S2 Table) and quantile-quantile plots (S1 Fig) are provided.

Differentially expressed microRNAs were analyzed using miRDB, an ontology network that captures microRNA and gene target interactions [25]. We also searched PubMed for all English-language manuscripts with the microRNA of interest as a search parameter. We focused on those manuscripts that examined relations between microRNA expression with processes involved in cardiac structural or electrophysiological remodeling, including cell-cell signaling, ion channels, myocardial fibrosis, inflammation, cardiomyocyte hypertrophy. All statistical analyses were performed using SAS (v9.4, SAS Institute Inc., Cary, North Carolina, USA).

## Results

The baseline demographic, clinical, and echocardiographic characteristics of the 1788 study participants are outlined in Table 1. Study participants were middle aged to older adults (mean age 66.4 years); over half (N = 972, 54%) were women. Two out of three (N = 1198, 67%) participants had a history of hypertension and (N = 85, 5%) had a history of AF.

**Table 1:** Characteristics of FHS Offspring study participants included in the analytic sample.

Variable*	Study
	Participants (n = 1788)
**Age, years**	66±9
**Sex (Female)**	972 (54%)
**Race (White)**	1788 (100%)
**Body mass index, kg/m**^**2**^	28±5
** Height (cm)**	167±10
** Weight (kg)**	79±17
**Current smoker**	173 (10%)
**Systolic blood pressure, mm Hg**	129±17
**Diastolic blood pressure, mm Hg**	73±10
**Antihypertensive medication use**	923 (52%)
**Diabetes mellitus**	269 (15%)
**Heart failure**	27 (2%)
**Stroke or transient ischemic attack**	68 (4%)
**Myocardial Infarction**	95 (5%)
**Atrial fibrillation**	84 (5%)
Incident atrial fibrillation**	157 (9%)

*All variables are reported as mean ± SD or N (%).

**Incident atrial fibrillation is reported for the participants who were free from atrial fibrillation at baseline.

When we examined the distribution of correlations from all microRNAs included in our analyses, we observed that 25% were strongly correlated (with correlation coefficients of 0.738 or higher, (S1 Table). Strong correlations between microRNAs may explain why a higher than expected number of microRNAs remained associated with LAFI after multivariable adjustment and correction for multiple testing using stringent criteria.

### Associations of microRNAs with AF

**Fig 2:**
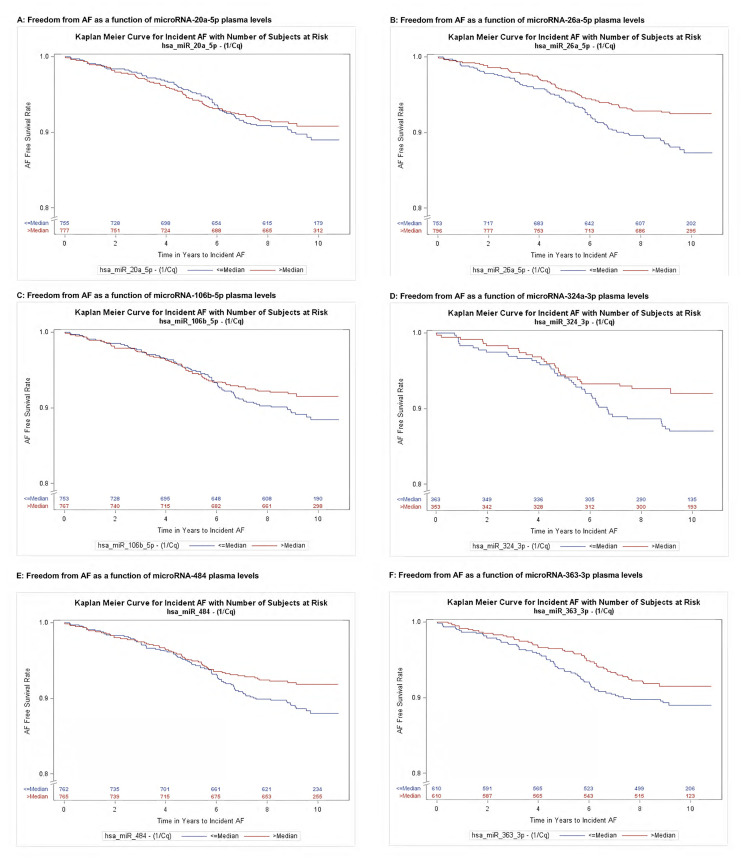
(A-F). Kaplan-Meier Plots show time to incident AF as a function of 6 microRNA plasma levels. Participants with lower than median microRNA plasma concentrations (1/C_q_) tended to have earlier onset of atrial fibrillation. Blue = Less than median plasma level. Red = Greater than median plasma level. Subfigures A-F show Kaplan-Meier plots for each identified microRNA.

LAFI-associated microRNAs (n = 73 microRNAs) were investigated for their relationships with prevalent AF using logistic regression models, and with incident AF using Cox regression models. Eighteen LAFI-associated microRNAs were also associated with prevalent AF (Table 2). Six were associated with incident AF, two of which were associated with incident AF alone (324-3p and 363-3p), whereas four microRNAs (106b, 26a-5p, 484, 20a-5p) were associated with both incident and prevalent AF (Table 3). Lower plasma levels of these six correlated with higher incidence of AF. Furthermore, among the six microRNAs associated with incident AF (except 363-3p), higher plasma levels correlated with greater LAFI and lower AF risk. Kaplan-Meier plots for time to incident AF are shown in Fig 2A–2F.

**Table 2:** Associations between and prevalent atrial fibrillation and plasma levels of LAFI-associated plasma microRNA.

	Prevalent AF Cases					Non-AF Cases								
MicroRNA	N	Median C_q_	Q1 C_q_	Q3 C_q_	Expression Mean C_q_	N	Median C_q_	Q1 C_q_	Q3 C_q_	Expression Mean C_q_	Hazard Ratio	95% CI		P-Value
**miR_20a_5p**	74	17.33	16.46	18.19	17.39	1532	16.84	16.04	17.63	16.87	1.36	1.14	1.61	0.001
**miR_106b_5**	74	18.16	17.36	19.09	18.23	1520	17.75	16.92	18.5	17.74	1.36	1.13	1.63	0.001
**miR_126_5p**	78	17.01	16.46	17.87	17.2	1575	16.75	16.03	17.48	16.8	1.32	1.1	1.58	0.003
**miR_26a_5p**	75	17.7	17.01	18.54	17.76	1549	17.3	16.51	18.15	17.35	1.28	1.07	1.54	0.007
**miR_15b_5p**	74	17.17	16.72	18.14	17.36	1544	16.97	16.29	17.66	17	1.31	1.08	1.59	0.007
**miR_484**	74	18.22	17.59	19.04	18.29	1527	17.92	17.12	18.64	17.9	1.3	1.07	1.58	0.007
**miR_93_5p**	72	18.27	17.49	19	18.25	1517	17.89	17.1	18.64	17.87	1.28	1.06	1.55	0.011
**miR_150_5p**	75	17.53	16.5	18.69	17.71	1533	17.01	16.27	18.21	17.28	1.21	1.04	1.41	0.013
**miR_30a_5p**	77	17.19	16.43	17.91	17.27	1554	16.85	16.15	17.55	16.91	1.25	1.05	1.49	0.013
**miR_140_3p**	69	19.61	18.73	20.35	19.55	1454	19.18	18.48	19.98	19.21	1.31	1.05	1.63	0.015
**miR_199a_3**	70	18.92	18.35	19.94	19.02	1489	18.64	17.84	19.54	18.67	1.26	1.04	1.53	0.019
**miR_17_5p**	73	18.21	17.34	18.9	18.17	1518	17.81	17.05	18.57	17.83	1.24	1.03	1.5	0.023
**let_7d_5p**	66	20.13	19.25	20.81	19.89	1292	19.58	18.71	20.37	19.55	1.29	1.04	1.6	0.023
**miR_23b_3p**	68	19.53	18.9	20.54	19.71	1427	19.35	18.63	20.2	19.41	1.28	1.02	1.6	0.032
**let_7b_5p**	61	19.96	19	20.64	19.82	1323	19.49	18.66	20.36	19.48	1.27	1.02	1.59	0.034
**miR_186_5p**	65	19.77	19	20.44	19.75	1396	19.48	18.7	20.23	19.44	1.28	1.02	1.61	0.340
**miR_27b_3p**	61	19.45	18.91	20.41	19.52	1333	19.04	18.26	20.11	19.17	1.23	1.01	1.51	0.041
**miR_19a_3p**	74	16.07	15.28	16.89	16.12	1539	15.75	14.96	16.55	15.8	1.18	1	1.39	0.048

*Expression levels are reported in C_q_ (inversely related to plasma levels). Note that hazard ratio described the risk in AF with 1 C_q_ increase.

**Table 3:** Associations between and incident atrial fibrillation and plasma levels of LAFI-associated plasma microRNA.

	Prevalent AF Cases					Non-AF Cases								
MicroRNA	N	Median Cq	Q1 Cq	Q3 Cq	Expression Mean Cq	N	Median Cq	Q1 Cq	Q3 Cq	Expression Mean Cq	Hazard Ratio	95% CI		P-Value
**miR_324_3p**	71	18.21	17.75	20.69	19.05	645	17.95	17.59	19.82	18.59	1.19	1.03	1.36	0.016
**miR_26a_5p**	141	17.62	16.68	18.4	17.54	1408	17.29	16.51	18.14	17.33	1.16	1.02	1.32	0.026
**miR_106b_5p**	139	17.99	16.93	18.7	17.93	1381	17.75	16.94	18.51	17.72	1.16	1.02	1.33	0.029
**miR_363_3p**	112	19.55	18.78	20.59	19.69	1108	19.29	18.59	20.24	19.43	1.17	1.01	1.36	0.043
**miR_484**	141	18.21	17.23	18.99	18.09	1386	17.91	17.13	18.64	17.89	1.15	1	1.32	0.049
**miR_20a_5p**	141	16.93	16.13	19.01	0.05	0.05	0.05	0.05	0.05	0.05	0.05	0.05	0.05	0.05

*Expression levels are reported in C_q_ (inversely related to plasma levels). Note that hazard ratio describes the risk in incident AF with 1 C_q_ increase

### Gene targets of microRNAs associated with AF

**Fig 3:**
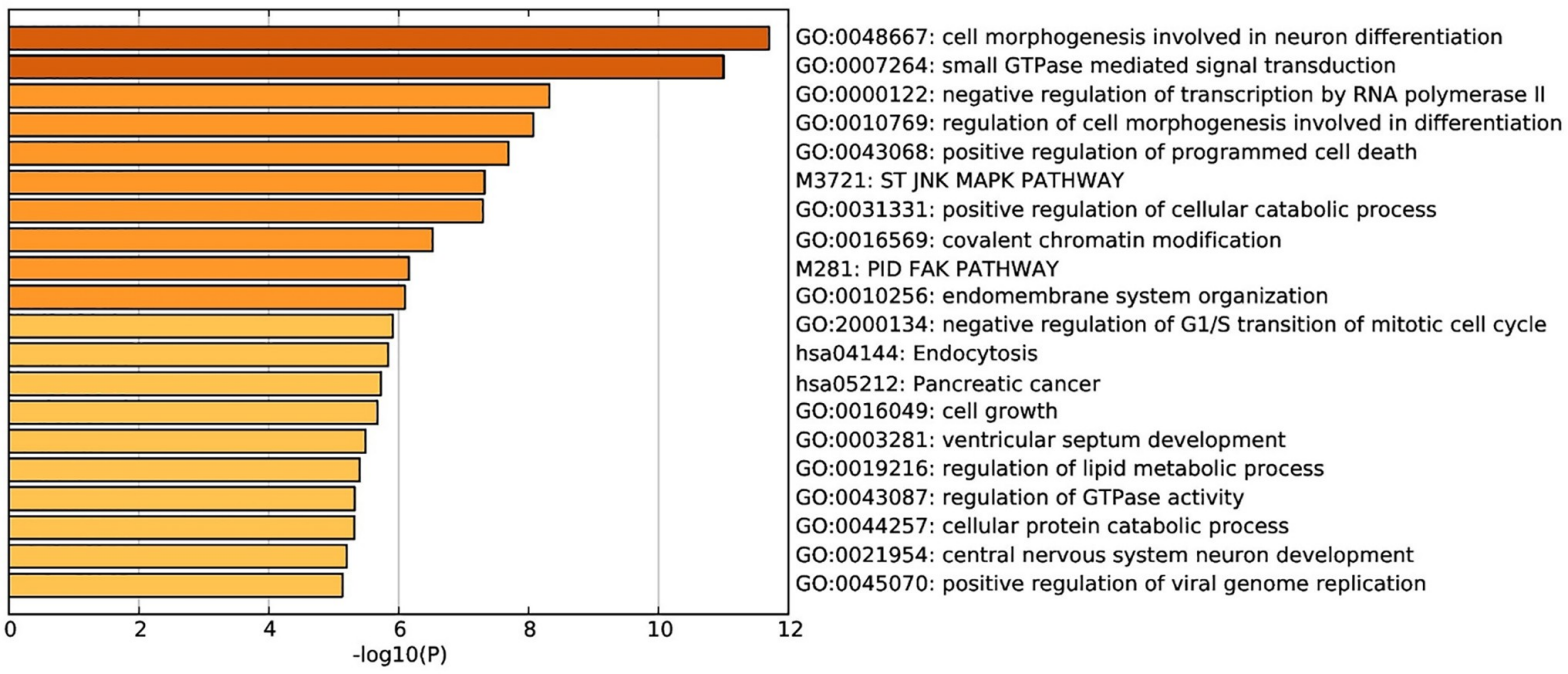
miRDB enrichment analysis conducted in metascape of six microRNAs associated with incident AF and. Each bar is labeled with associated process. The length of each bar is the negative base-10 logarithm of the calculated p-value based on a cumulative hypergeometric distribution.

We investigated potential targets of the six microRNAs associated with incident AF and LAFI through miRDB. 2402 genes were predicted as targets for at least one microRNA, among which 939 genes were predicted as targets for at least two microRNAs. Fig 3 shows the results of an enrichment analysis performed using Metascape [26]. The microRNA-related genes were significantly enriched into 20 categories with the two most significant being related to cell morphogenesis (GO0048667) and signal transduction (GO0007264). Furthermore, a comprehensive search of gene ontology databases and the published literature identified several genes known to influence susceptibility to incident AF that were regulated by the microRNAs identified in our analyses (Table 4).

**Table 4:** Known gene targets and phenotypes from gene ontology of the 6 microRNAs associated with LAFI and incident AF.

MicroRNA	Expression levels in relation to LAFI	Expression Levels in Relation to AF Risk	Function (Target Genes) *	Associated Phenotype*
**20a-5p**	↑	↓	Smoothened (SMO) [27]	Cell Proliferation; Apoptosis; Hh signaling pathway
**26a-5p**	↑	↓	Potassium Voltage-Gated Channel Subfamily J Member 2 (KCNJ2), Cyclins (CCND2, CCNE1, CCNE2 and CDK6) [28, 29, 30]	Altered inward-rectifier potassium channel function; AF; Suppression of G1/S phase transition
**106b-5p**	↑	↓	Short Stature Homeobox 2 (Shox2), T-Box 3 (Tbx3) [16]	Atrial arrythmias; Sinoatrial dysfunction
**324-3p**	↑	↓	Dynein Cytoplasmic 1 Light Intermediate Chain 2 (DYNC1LI2), Ankyrin Repeat and Sterile Alpha Motif Domain Containing 1A (ANKS1), ATP Binding Cassette Subfamily G Member 1 (ABCG1), Cytohesin 3 (CYTH3), Apoptosis Resistant E3 Ubiquitin Protein Ligase 1 (AREL1) [19]	Cytoskeletal remodeling; cellular signaling; Apoptosis
**484**	↑	↓	Mitochondrial Fission protein (Fis1) [31]	Decreased Cardiac myocyte apoptosis
**363-3p**	↓	↓	DnaJ Heat Shock Protein Family (Hsp40) Member B9 (DNAJB9), Solute Carrier Family 12 Member 5 (SLC12A5), F-Box and WD Repeat Domain Containing 7 (FBXW7) [21]	Apoptosis; Ion channels; proteosomal degredation

*The gene targets and associated phenotypes for each of the 6 microRNAs associated with AF were obtained from publicly available gene ontology databases (Methods) of animal and human studies or from a search of English-language peer-reviewed manuscripts. See Methods section for full details

## Discussion

In our investigation of Framingham Offspring Study Cohort Exam 8 participants, we identified 73 plasma microRNAs associated with LAFI (S3 Table), a validated marker of structural and functional left atrial remodeling [9]. We identified 20 microRNAs associated with LAFI and either prevalent or incident AF. Our investigation validates previously observed associations between plasma microRNAs and prevalent AF and identifies new associations with incident AF in a community-based sample. Further, our study may provide potentially important mechanistic insights by demonstrating associations between common microRNAs, LAFI, and AF (Fig 3, Table 4). Our findings suggest that adverse structural LA remodeling, as measured by LAFI, may be influenced by, or related to, the circulating transcriptome and thereby contribute to risk for AF.

### LAFI as an intermediate cardiac phenotype predisposing to AF

Larger mean LA size and lower mean LA phasic function are two validated echocardiographic markers of adverse LA structural and functional remodeling [8]. Recent data from large community-based samples demonstrate the importance of LA phasic function, adjusting for LA volume, as an intermediate cardiac phenotype predisposing to AF [9]. Composite measures of LA size and function, such as LAFI, may be more sensitive to detecting adverse LA remodeling than LA volume or emptying fraction alone. Measures of structural and functional LA remodeling correlate with histological evidence of cellular hypertrophy as well as extracellular collagen deposition, metabolic dysregulation, and myocyte cell death [32,33]. Furthermore, a decrease in atrial phasic function relates strongly to altered calcium-handing and ion channel dysregulation (i.e., L-type Ca^2+^ channels and Na^+^/Ca^2+^exchanger) in myocytes. Therefore, the structural and functional LA remodeling, as measured by lower LAFI, may also capture some aspects of adverse electrical LA remodeling, making LAFI a suitable quantitative cardiac phenotype to identify microRNAs that might be associated with AF. Our decision to choose LAFI as the intermediate phenotype was based on the observations that AF predominantly affects older adults, relates strongly to duration and intensity of exposure to risk factors, and is frequently preceded over mid-adulthood by the development of an intermediate phenotype, echocardiographic atrial remodeling. This also incorporates patients with valvular defects, as valvular defects would alter (LVOT-VTI) measurements contributing to increased incidence of LAFI and ultimately AF through atrial remodeling.

### Association of microRNAs with LA remodeling

In our previous work [12], we chose to focus on risk factors related to degree of pathological atrial remodeling, which included hypertension, heart failure, and myocardial infarction which are potential mediators of atrial remodeling and AF vulnerability. Thus, in this study, we chose to focus on the echocardiographic findings of structural remodeling that lead to AF (i.e. LAFI) because it is an established measure of atrial mechanical function and is strongly associated with risk for incident AF.

The association of microRNAs with LA structural remodeling has been explored in canine models with AF and rat models with myocardial infarction. In prior studies, up-regulation of microRNA-21 and downregulation of microRNAs 26, 29b, 30, 133, and 590 in atrial tissues has been associated with increased levels of fibrosis mediators such as TGF-β1 and TGFR-2 and histological evidence of left atrial extracellular fibrosis [34,35]. However, few prior studies have examined quantitative echocardiographic traits in humans in relation to cardiac or plasma microRNA expression. We identified 73 microRNAs with statistically significant associations with LAFI after adjusting for CHARGE-AF risk factors (S3 Table), including microRNAs 21, 26, 29b and 30. Most LAFI-associated microRNAs (n = 61) showed positive associations (lower levels, lower LAFI). Many of these microRNAs regulate cytoskeletal remodeling, ion channel function, cardiac fibrosis, myocyte apoptosis, and cardiac hypertrophy in human or animal models [36–38]. Our findings suggest the macroscopic LA remodeling, as captured by LAFI, is closely associated with the circulating transcriptome, even after adjustment for known clinical and electrocardiographic associates of LA remodeling and AF.

### Association of microRNAs with AF

Twenty plasma microRNAs were significantly associated with *both* LAFI and AF after adjustment for covariates and correction for multiple testing (Tables 2 and 3). While several microRNAs identified in the present analysis have previously been associated with AF in experimental models, our results should also be viewed in the context of prior small-to-intermediate sized cross-sectional human and experimental studies examining relations between cardiac, whole-blood, platelet, or plasma microRNAs with AF [12,39,31]. Although we identified a different set of microRNAs associated with AF than we observed in our prior study examining whole blood microRNA levels and AF in the Framingham cohort, significant overlap was observed between the findings of this analysis and results from our prior investigation examining plasma microRNA levels and AF in a prospectively recruited cohort of 112 AF patients and 99 referents [7,12]. For example, microRNAs 150, and let-7b were associated with prevalent AF in both investigations [7]. Differences in the results of this study as compared with our prior investigation likely result from the current study’s longer duration of follow up, and known differences in microRNA pools present in plasma and whole-blood because the whole blood miRNome includes microRNAs in white and red blood cells, as well as platelets [12,40]. Our results are consistent with prior work, lending confidence to our approach and findings, but also extend our observations to a larger community-based cohort.

In contrast to prior work, our present analysis was designed and powered sufficiently to identify plasma microRNAs associated with incident AF. Of the six microRNAs associated with incident AF, four were also associated with prevalent AF in separate analyses, suggesting shared gene regulatory networks. Our ontological and enrichment analyses and review of the literature (Fig 3, Table 4) demonstrate that of the six microRNAs associated with LAFI and incident AF, four (microRNAs 106b-5p, 26a-5p, 324a-3p, 20a-5p) have established roles in the pathophysiology of atrial remodeling and AF, whereas two (microRNAs 363-3p, 484), represent novel discoveries with plausible relations to AF (Table 4). MicroRNAs associated with AF in our analyses have strong quantifiable associations with genes involved in cell morphogenesis (GO004667), signal transduction (GO0007264), cell death, and mitotic transition (GO0043068, GO2000134), and as well as cellular differentiation and catabolic processes (Fig 3). Interestingly, cellular morphogenesis in GO004667 relates to neuronal differentiation, and we speculate that microRNAs may exert their influence on AF vulnerability by regulating genes involved in the autonomic nervous system. This discovery highlights the potential interactions between cardiac structural remodeling, epicardial adipose tissue, and the extensive network of vagal plexi known to affect risk for AF [40].

The Kaplan Meier curves for microRNAs 106b, 324, 484 diverge after nearly 6 years of follow-up, whereas those for microRNAs 26a and 363 separated from the time of transcriptomic profiling (Fig 2). We submit that these findings support an association for the microRNAs identified as being associated with AF. Atrial remodeling takes years to occur, precedes the development of AF, and is likely controlled by subtle perturbations in gene regulatory networks over time, as reflected by dysregulated microRNA expression [41]. Therefore, it would be expected for some microRNAs to take years to affect an intermediate phenotype (lower LAFI) or end-stage phenotype (AF). Future studies should leverage multiple-time microRNA data collection for each participant, to test both these hypotheses.

### Strength and limitations

Our study had several notable strengths. Our study profiles plasma microRNA expression in the largest sample of community-based participants to date using highly sensitive and specific PCR methods that have excellent discriminative ability. We leveraged rigorously adjudicated clinical and echocardiographic data from a representative sample of participants enrolled in the community-based Framingham Offspring Study and our statistical analyses adjusted for clinical and electrocardiographic correlates of LA remodeling and AF. We used a conservative Bonferroni correction at Step 1. Furthermore, the microRNAs observed to be in association with AF are highly consistent with respect to the strength of statistical associations and the directionality of associations with AF when compared to prior work involving separate cohorts. In addition, our study incorporates measures obtained from a longer participant follow up and captures more cases of incident AF.

Our study does have several limitations. First, because our study sample was comprised of participants largely of European-American ancestry, the generalizability of our findings to individuals of other races/ethnicities is uncertain. Secondly, we did not control for medications in this study owing to the study sample size although to our knowledge, there have been no trials demonstrating the effects of specific cardiovascular or non-cardiovascular drugs that influence plasma concentrations of the 20 microRNA associated with prevalent or incident AF. Furthermore, although the low incidence and prevalence of AF in our study is consistent with rates seen in other cohorts, we acknowledge that AF can be paroxysmal and elude clinical detection [2]. Thirdly, as LAFI is mathematically determined from LVOT-VTI, presence of valvular disease may independently affect LAFI and make it less reliable. The specific effect is unknown and future investigations should compare LAFI with more traditional measures of LA function, in patients with valvular disease. Additionally, although our group and others have demonstrated strong relations between cardiac microRNA and plasma microRNA expression profiles in patients with AF and other types of CVD, cardiac tissue was not available from FHS Offspring participants and thus we could not compare the cardiac and circulating transcriptome [6,12, 41]. Based on our prior data, we strongly suspect that the plasma miRnome relates to cardiac gene regulation and microRNA expression, but longitudinal studies which incorporate a mediation analysis are needed to establish this relationship. Our study was observational; we cannot rule out residual confounding and we cannot establish causal relations between the microRNAs, LAFI, and AF.

### Conclusion

In our study including 1788 FHS Offspring participants with available echocardiographic, clinical, and microRNA data, we observed that several microRNAs known to regulate genes implicated in cardiac fibrosis, inflammation, and myocyte apoptosis were associated with LAFI and AF. Our findings contribute to emerging literature consistent with the hypothesis that circulating microRNAs play a critical role in the pathophysiology of atrial remodeling and fibrillation.

## Supporting information

S1 Text Methodology of study. (PDF)

S2 Text Literature review of mechanisms of microRNA-mediated atrial fibrillation. (PDF)

S1 Table Distribution of correlations from all micro RNAs. (TIFF)

S2 Table Pearson partial correlation coefficient with LAFI controlling for CHARGE-AF. (PDF)

S3 Table Strength and directionality of statistically significant association between 73 plasma microRNA levels and echocardiographic left atrial function index. (TIFF)

S4 Table Non-significant associations between and prevalent atrial fibrillation and plasma levels of LAFI-associated plasma microRNA. (PDF)

S1 Fig Quantile-Quantile Plots all Micro RNAs. (TIFF)

S1 File (DOCX)
