# Supplementary material for: Relations between plasma microRNAs, echocardiographic markers of atrial remodeling, and atrial fibrillation: Data from the Framingham Offspring study
Source: PLoS One. 2020 Aug 19;15(8):e0236960. doi: 10.1371/journal.pone.0236960 (PMC7437902; doi:10.1371/journal.pone.0236960)
Supplement: S1 Text — (PDF) [file pone.0236960.s001.pdf]

Supplement 1: Methodology

Blood sample collection and plasma separation:
Blood samples were previously collected at Framingham Heart Study (Framingham, MA, USA) on Offspring exam 8 (March 2005–Jan 2008). Venipuncture was performed on study participants in a supine position after an overnight fast, using standard venipuncture techniques. Blood was collected into blood collection tubes with a liquid buffered sodium citrate additive (0.105 M). Blood collection tubes were centrifuged at 2,500g for 22 min at 4°C. Plasma was separated from the cells and frozen at 80°C within 90 min of draw. An aliquot of 170 µl of plasma samples was transferred to our laboratory in March 2014 and stored at 80 C. Freezers were both generator and CO_2 backed up.

RNA isolation from plasma:
RNAs was isolated from plasma samples using a miRCURY RNA Isolation Kit—Biofluids (Cat. No: 300112, Exiqon, Denmark). A total of 130 ml of plasma samples were used in each RNA isolation. The kit is designed to isolate RNA from 200 ml of plasma. We replaced the remaining volume with nuclease-free water. Manufactures protocol was followed with one modification that was reducing the elution volume from 50 µl nuclease-free water to 30 µl. After thawing the plasma samples tubes were mixed by inverting. Then plasma samples were centrifuged at 8,000g for 5 min. One-hundred thirty µl of plasma samples from the top of the tubes transferred into a new 2.0 ml DNA LoBind tubes (Cat. No: 022431048, Eppendorf, Germany) and 70 µl nuclease free water added to each sample to complete the volume to 200 µl. Sixty µl Lysis Solution BF were added to each sample. Tubes were vortexed for 15 s. Tubes were incubated for 5 min at room temperature. Twenty µl Protein Precipitation Solution BF were added into each tube and tubes were vortexed for 15 s. Vortexed tubes were incubated for 1 min at room temperature. Tubes were centrifuged for 3 min at 16,000g. Clear supernatants were transferred into a clean 2.0 ml DNA LoBind tubes. Two-hundred seventy µls of Isopropanol was added into each tubes and tubes were vortexed for 5 s. Tubes were centrifuged briefly (2–3 s) to collect all liquid at the bottom of the tubes. MicroRNA Mini Spin Columns BFs connected to vacuum manifold called QIAvac 24 Plus (Cat. No: 19413, Qiagen, Germany) by using VacValves (Cat. No: 19408, Qiagen, Germany). Samples were transferred into microRNA Mini Spin Columns BFs using transfer pipettes. Samples were incubated for 2 min at room temp on microRNA Mini Spin Column BFs with open lids. Vacuum pump was turned on until all liquids were passed. Seven hundred µl of Wash Solution 2 BF (80 ml Absolute ethanol was added to Wash Solution 2 BF bottle) was added on microRNA Mini Spin Column BFs and vacuum pump was turned on until all liquids were passed. Two-hundred fifty µl of Wash Solution 2 BF was added on microRNA Mini Spin Column BFs and vacuum pump was turned on until all liquids were passed. Hundred µl Wash Solution 1 BF was added on microRNA Mini Spin Column BFs and vacuum pump was turned on until all liquids were passed. Seven hundred µl of Wash Solution 2 BF was added on microRNA Mini Spin Column BFs and vacuum pump was turned on until all liquids were passed. Two-hundred fifty µl Wash Solution 2 BF was added on microRNA Mini Spin Column BFs and vacuum pump was turned on until all liquids were passed. MicroRNA Mini Spin Column BFs were transferred to collection tubes (2.0 ml without a lid). Tubes were centrifuged for 2 min at 11,000g at room temperature to dry the membranes completely. Centrifuged microRNA Mini Spin Column BFs were transferred into 1.5 ml DNA LoBind tubes (conical bottom tubes) and 30 µl of nuclease-free water added directly onto membranes. Tubes were incubated for 1 min at room temp with lids open. Lids were closed and centrifuged for 1 min at 11,000g at room temperature. RNA samples were transferred into the V bottom, snap cap, 0.5 ml micronic tubes in two aliquots (12.5 µl in each) and capped. 2D barcodes on the tubes were

recorded and RNA samples were kept at 80 C. RNA samples randomly analyzed on Bioanalyzer 2100 by using RNA 6000 Pico Kit (Agilent Technologies, Santa Clara, CA).

Standardization of sample volume:

Throughout the experiments, we consistently used 5 µl of RNA samples (isolated from 130 ml of plasma) for all of the samples. A fixed volume of plasma from each participant in the RT-qPCR experiment workflow was used and the same volume of RNA elution (30 µl) was used for all isolations keeping these variables constant across all samples. Also, RNA volumes for reverse transcription reactions (5 µl) were constant for each sample. The same volume (5 µl) of 1:5 diluted cDNA were used in each preamplification reaction. Two µl of 1:5 diluted preamplified cDNA samples were used in each qPCR experiment. By doing this we minimized the variance from sample to sample for possible variance for the amount of RNA. Another reason we used a fixed volume based approach is the lack of consensus in regards to housekeeping small-RNAs for extracellular compartments such as plasma.

Reverse Transcription: RNA samples were reverse transcribed by using miScript II RT Kit (Cat. No: 218161, Qiagen, Fredrick, MD, USA). RNA samples were thawed on ice. Reverse transcription master mix was prepared by combining 950 µl of 5 miScript HiSpec Buffer, 475 µl of 10 miScript Nucleics Mix, 475 µl of nuclease-free water, and 475 ml of miScript Reverse Transcriptase Mix for a total volume of 2,375 µl, which was enough for four sets of 96 samples. Five µl of this master mix were dispensed into each well of a 96-well PCR plate (Cat. No: AB17500, Bioplastic, Netherland). Five µl of RNA samples were then transferred into each well by using Vioflo384 Pipetting System (Integra Biosciences, Hudson, NH, USA) with 96-well head. Plates were sealed with EU optical Wide 8-Cap Strip Mats (Cat. No: B57651, Bioplastic, Netherland), vortexed on a plate shaker for 10 s by pressing with hands and then centrifuged for 1 min at 500g in a small-plate centrifuge. The samples were then incubated at 37°C for 60 min and then at 95°C for 5 min in the ProFlex 96 block Thermal Cycler (Life Technologies, USA). When the reverse transcription reactions were completed, plates were centrifuged for 1 min, the strip mats covering was removed and 40 µl of nuclease-free water were added into each well by using Vioflo384 Pipetting System (Integra Biosciences, Hudson, NH, USA) with 96-well head. Plates were again sealed with EU optical Wide 8-Cap Strip Mats (Cat. No: B57651, Bioplastic, Netherland), vortexed on a plate shaker for 10 s by pressing with hands and then centrifuged for 1 min at 500g in a small-plate centrifuge.

Preamplification of cDNAs: miScript Microfluidics PreAMP Kit (Cat. No: 331455, Qiagen, Fredrick, MD, USA) was used for preamplification reactions. Manufacturer instructions were followed with a modification to reduce the preamplification reaction final volume from 25 µl to 15 µl. Preamplification master mix was prepared on ice by combining 1,425 µl of 5X miScript PreAMP Buffer, 570 µl of HotStarTaq DNA Polymerase, 1,425 µl of miScript PreAMP Primer Mix, 1,995 µl of nuclease-free water and 285 µl of miScript PreAMP Universal Primer (10 µM) for a total volume of 5,700 µl, which was enough for 4 sets of 96 samples. 12 ml of this master mix was dispensed into each well of a 96-well PCR plate (Cat. No: AB17500, Bioplastic, Netherland). Three µl of diluted cDNA samples were transferred into each well by using Vioflo384 Pipetting System (Integra Biosciences, Hudson, NH, USA) with 96-well head. Plates were sealed with EU optical Wide 8-Cap Strip Mats (Cat. No: B57651, Bioplastic, Netherland), vortexed on a plate shaker for 10 s and then centrifuged for 1 min at 500g in a small-plate centrifuge. The samples were then cycled in a ProFlex 96 block Thermal Cycler (Life Technologies, USA) as follows: one cycle of the PCR initial activation step for 15 min at 95 C, followed by two cycles of denaturation for 30 s at 94°C, annealing for 60 s at 60°C and extension for 60 s at 60°C, followed by 10 cycles of denaturation for 30 s at 94°C, and annealing/ extension for 3 min at 60°C. When preamplification reactions were completed the

plates were centrifuged for 1 min, the strip mats covering was removed and 2 µl of SR1 (side reaction reducer ¼ Exonuclease I) was added into each well by using Vioflo384 Pipetting System (Integra Biosciences, Hudson, NH, USA) with 96-well head. Plates were then sealed again with EU optical Wide 8-Cap Strip Mats (Cat. No: B57651, Bioplastic, Netherland), vortexed on a plate shaker for 10 s and then centrifuged for 1 min at 500g on a small-plate centrifuge. Samples were then cycled in at ProFlex 96 block Thermal Cycler (Life Technologies, USA) as follows: 37°C for15 min; and 95°C for 5 min. When SR1 reactions were completed, the plates were centrifuged for 1 min, the strip mats covering was removed and 58 µl of nuclease-free water was added into each well by using Vioflo384 Pipetting System (Integra Biosciences, Hudson, NH, USA) with 96-well head. Plates were then sealed again with EU optical Wide 8-Cap Strip Mats (Cat. No: B57651, Bioplastic, Netherland), vortexed on a plate shaker for 10 s and then centrifuged for 1 min at 500g on a small-plate centrifuge.

Real-Time PCR miRNA Profiling: Assay plates and preamplification primer mix preparation was performed according to the miScript Microfluidics Handbook (https://www.qiagen.com/us/resources/resourcedetail?id=34798505-6ff7-459c- 8896-2ee777093f6e&lang=en)). miRNA Assays were purchased from Qiagen in dried down format. First, assay plates were centrifuged at 1,500g for 10 min. Then, 27.5 µl of nuclease-free water was added into each well and incubated at room temperature for 10 min. Plates were vortexed in a plate shaker for 3 min at room temperature. These assay were at 100 µM concentrations. Eighteen µl of these assays were taken and combined in a reservoir (43 snoRNA assays were added separately, see below) and 2,106 µl of nuclease-free water was added to make a preamplification primer pool. 14.25 µl of nuclease-free water was added onto the remaining 9.5 ml of 100 µM assays for a total volume of 23.75 µl and final concentration of 40 µM. Equal volume of miScript Microfluidics Universal Primer (40 mM) was added onto these assays for a total volume of 47.5 µl and final assay concentration of 20 µM. Equal volume of the 2X Assay Loading Reagent (Cat. No: 100-7611, Fluidigm, South San Francisco, CA, USA) (47.5 ml) was added onto these assays. The assays were now ready to load into Dynamic Arrays. 28 µl aliquots of these assays were aliquoted in 96-well Piko plates (Cat. No: SPL0960, Fisher Scientific, USA), sealed and stored at 20°C. snoRNA assays and piRNA assays were custom designed by Qiagen and synthesized primers were delivered at 100 µM concentration in liquid form. These assays were also prepared as described above.

Dynamic Array Preparations for qPCR: Control line fluid was injected into appropriate positions on two Dynamic Arrays 96.96 GE and the Dynamic Arrays were placed into IFC Controller HX for priming. While priming was performed (20 min), qPCR master mix was prepared on ice by combining 720 µl of Microfluidics qPCR Master Mix (Qiagen), 72 µl of 20 DNA Binding Dye Sample Loading Reagent (Fluidigm, PN 85000746), and 168.00 µl of nuclease-free water for a total of 960 µl, which was enough for two sets of 96 samples. Four µl of this master mix were dispensed into each well of a 96-well PCR plate (Cat. No: AB17500, Bioplastic, Netherland). Two µl of diluted preamplified cDNA samples were then transferred into each well using Vioflo384 Pipetting System (Integra Biosciences, Hudson, NH, USA) with 96-well head. Plates were then sealed with EU optical Wide 8-Cap Strip Mats (Cat. No: B57651, Bioplastic, Netherland), vortexed on a plate shaker for 10 s and then centrifuged for 1 min at 500g on a small-plate centrifuge. Five µl of this mixture were aspirated by using Viaflo 8-channel electronic pipet and dispensed into sample inlets of Dynamic Arrays as 4.75 µl to avoid creating bubbles. 4.75 ml of assays were aspirated by using Viaflo 8-channel electronic pipet and dispensed into sample inlets of Dynamic Arrays as 4.50 µl to avoid creating bubbles. Dynamic Arrays were placed into the IFC Controller HXs and the standard Load script was started (B90 min). When the loading script was finished, dynamic arrays were loaded into the BioMark reader and cycling was performed as follows: thermal mix for 2 min at 50°C; 30 min at 70°C; and 10

min at 25°C, followed by PCR initial activation step for 10 min at 95°C, followed by a three-step cycling for 23 cycles of denaturation for 15 s at 94°C, annealing for 30 s. at 55°C and extension for 30 s. At 70°C, followed by performing fluorescence data and collection and dissociation curve analysis. ROX was used as a passive reference dye. Linear derivative and user global setting (Threshold setting 0.002) were used as setting parameters, samples and assays names were entered and basic real-time PCR analysis was performed on BioMark Real-Time PCR Analysis software. Quantification cycle (Cq) values were exported as csv files.
