# Supplementary material for: Relations between plasma microRNAs, echocardiographic markers of atrial remodeling, and atrial fibrillation: Data from the Framingham Offspring study
Source: PLoS One. 2020 Aug 19;15(8):e0236960. doi: 10.1371/journal.pone.0236960 (PMC7437902; doi:10.1371/journal.pone.0236960)
Supplement: S2 Table — (PDF) [file pone.0236960.s004.pdf]

Supplemental Table 5: Pearson Partial Correlation Coefficient with LAFI controlling for CHARGE-AF

MiRNA	Pearson Partial Correlation Coefficient	P-value
hsa_miR_128_3p	0.14246	<0.001
hsa_let_7i_5p	-0.17156	<.0001
hsa_miR_191_5p	0.18802	<.0001
hsa_miR_494_3p	0.13549	<.0001
hsa_miR_195_5p	-0.28116	<.0001
hsa_miR_32_5p	0.16859	<.0001
hsa_miR_652_3p	0.16178	<.0001
hsa_miR_21_5p	-0.1616	<.0001
hsa_miR_363_3p	0.13074	<.0001
hsa_miR_28_3p	-0.1824	<.0001
hsa_miR_1246	-0.24904	<.0001
hsa_miR_421	-0.24687	<.0001
hsa_miR_19a_3p	-0.14851	<.0001
hsa_miR_126_5p	-0.15195	<.0001
hsa_miR_194_5p	0.28061	<.0001
hsa_miR_150_5p	-0.25326	<.0001
hsa_miR_324_3p	-0.25889	<.0001
hsa_miR_532_3p	-0.16905	<.0001
hsa_miR_3613_3p	-0.19414	<.0001
hsa_miR_20a_5p	-0.16715	<.0001
hsa_miR_106b_3p	0.12598	<.0001
hsa_miR_126_3p	-0.10942	<.0001
hsa_miR_30a_5p	-0.10591	<.0001
hsa_miR_582_5p	-0.3314	<.0001
hsa_miR_142_5p	-0.14334	<.0001
hsa_miR_382_5p	-0.23538	<.0001
hsa_miR_15b_5p	-0.11384	<.0001
hsa_miR_323b_3p	-0.25725	<.0001
hsa_miR_199a_3p	-0.14096	<.0001
hsa_miR_320b	-0.29811	<.0001
hsa_miR_628_3p	0.11678	<.0001
hsa_miR_26a_5p	-0.17517	<.0001
hsa_miR_1301_3p	0.0953	0.024
hsa_miR_140_3p	-0.09379	0.0003
hsa_let_7b_5p	-0.18549	<.0001
hsa_miR_30e_5p	-0.11676	<.0001
hsa_miR_146a_5p	-0.10582	<.0001
hsa_miR_484	-0.12062	<.0001
hsa_miR_664b_3p	0.31684	<.0001

MiRNA	Pearson Partial Correlation Coefficient	P-value
hsa_miR_185_5p	-0.16814	<.0001
hsa_miR_301b_3p	0.18896	<.0001
hsa_miR_29a_3p	-0.10082	<.0001
hsa_miR_27a_3p	-0.25629	<.0001
hsa_miR_30c_5p	-0.13603	<.0001
hsa_miR_144_3p	-0.15048	<.0001
hsa_miR_92a_3p	-0.10374	<.0001
hsa_miR_106b_5p	-0.161	<.0001
hsa_miR_425_5p	-0.16623	<.0001
hsa_miR_130a_3p	-0.10097	<.0001
hsa_miR_30b_5p	-0.14845	<.0001
hsa_miR_17_5p	-0.16443	<.0001
hsa_miR_122_5p	-0.13477	<.0001
hsa_miR_451a	-0.16116	<.0001
hsa_miR_423_5p	-0.13999	<.0001
hsa_let_7a_5p	-0.16437	<.0001
hsa_miR_93_5p	-0.13591	<.0001
hsa_miR_29c_3p	-0.112	<.0001
hsa_miR_22_3p	-0.11767	<.0001
hsa_miR_23b_3p	-0.17033	<.0001
hsa_miR_148a_3p	-0.22124	<.0001
hsa_let_7g_5p	-0.15343	<.0001
hsa_miR_223_3p	-0.23396	<.0001
hsa_miR_26b_5p	-0.179	<.0001
hsa_miR_29b_3p	-0.13803	<.0001
hsa_miR_1260a	-0.20368	<.0001
hsa_miR_424_5p	-0.19014	<.0001
hsa_miR_125a_5p	-0.21517	<.0001
hsa_miR_23a_3p	-0.1552	<.0001
hsa_miR_148b_3p	-0.19381	<.0001
hsa_miR_27b_3p	-0.16455	<.0001
hsa_miR_186_5p	-0.09238	0.0005
hsa_let_7d_5p	-0.16326	<.0001
hsa_miR_25_3p	-0.19776	<.0001
