# Supplementary material for: Relations between plasma microRNAs, echocardiographic markers of atrial remodeling, and atrial fibrillation: Data from the Framingham Offspring study
Source: PLoS One. 2020 Aug 19;15(8):e0236960. doi: 10.1371/journal.pone.0236960 (PMC7437902; doi:10.1371/journal.pone.0236960)
Supplement: S4 Table — (PDF) [file pone.0236960.s006.pdf]

Supplemental Table 2: Non-significant associations between and prevalent atrial fibrillation and plasma levels of LAFI-associated plasma microRNA.

	PREVALENT AF CASES		NON-AF CASES					
**MICRORNA**	**N**	**EXPRESSION MEAN C_Q**	**N**	**EXPRESSION MEAN C_Q**	**OR**	**95% CI**		**P-VALUE**
**MIR_21_5P**	75	16.15	1551	15.85	1.18	1.00	1.39	0.056
**MIR_451A**	75	12.43	1549	12.01	1.11	1.00	1.25	0.058
**MIR_125A_5**	64	19.67	1347	19.42	1.23	0.98	1.55	0.069
**MIR_29A_3P**	74	17.16	1530	16.90	1.19	0.99	1.43	0.069
**MIR_26B_5P**	73	17.72	1545	17.46	1.18	0.98	1.42	0.079
**MIR_628_3P**	31	18.28	741	18.60	0.75	0.54	1.05	0.089
**MIR_191_5P**	75	16.29	1545	15.91	1.11	0.98	1.26	0.095
**MIR_532_3P**	48	19.35	1072	19.71	0.86	0.72	1.03	0.099
**MIR_29C_3P**	74	17.14	1530	16.91	1.17	0.97	1.42	0.104
**MIR_23A_3P**	74	16.15	1548	15.94	1.18	0.97	1.43	0.109
**MIR_223_3P**	74	15.12	1557	14.82	1.12	0.97	1.30	0.111
**MIR_425_5P**	54	19.83	1157	19.57	1.21	0.96	1.53	0.113
**MIR_142_5P**	66	18.99	1460	18.75	1.16	0.95	1.40	0.139
**MIR_30E_5P**	75	17.23	1546	17.03	1.16	0.95	1.42	0.139
**MIR_106B_3**	61	17.60	1193	17.95	0.90	0.78	1.04	0.144
**MIR_92A_3P**	75	15.57	1551	15.37	1.15	0.95	1.38	0.152
**MIR_30C_5P**	63	19.99	1317	19.79	1.20	0.94	1.53	0.155
**MIR_1301_3**	23	19.97	568	20.26	0.76	0.51	1.12	0.158
**LET_7G_5P**	55	19.74	1271	19.50	1.18	0.94	1.48	0.159
**MIR_194_5P**	68	18.14	1423	18.39	0.89	0.75	1.05	0.163
**MIR_130A_3**	60	19.75	1369	19.55	1.17	0.93	1.48	0.179
**MIR_25_3P**	75	17.19	1550	16.98	1.12	0.94	1.33	0.197
**MIR_146A_5**	70	18.61	1519	18.44	1.13	0.92	1.40	0.242

	PREVALENT AF CASES		NON-AF CASES					
MICRORNA	N	EXPRESSION MEAN C_Q	N	EXPRESSION MEAN C_Q	OR	95% CI		P-VALUE
MIR_652_3P	58	18.35	1216	18.68	0.93	0.82	1.05	0.244
MIR_323B_3	35	17.50	963	17.86	0.90	0.74	1.09	0.271
MIR_301B_3	53	18.50	977	18.22	1.09	0.94	1.26	0.280
MIR_421	74	16.68	1523	16.23	1.04	0.97	1.11	0.283
MIR_29B_3P	54	19.81	1279	19.65	1.15	0.89	1.47	0.286
MIR_27A_3P	73	17.76	1514	17.56	1.08	0.94	1.24	0.307
MIR_185_5P	66	19.20	1415	19.04	1.11	0.91	1.35	0.311
LET_7A_5P	66	19.64	1355	19.49	1.11	0.90	1.36	0.334
MIR_126_3P	75	16.26	1557	16.13	1.09	0.91	1.31	0.351
MIR_144_3P	56	19.24	1192	19.04	1.09	0.91	1.30	0.363
MIR_320B	52	17.35	1150	16.98	1.04	0.95	1.14	0.392
MIR_195_5P	73	17.07	1508	16.76	1.03	0.96	1.12	0.396
MIR_382_5P	60	18.84	1368	18.66	1.06	0.92	1.23	0.439
MIR_148B_3	69	18.71	1479	18.59	1.08	0.90	1.29	0.441
MIR_30B_5P	63	19.81	1355	19.71	1.10	0.86	1.40	0.445
MIR_3613_3	34	18.86	780	18.65	1.08	0.88	1.32	0.477
MIR_148A_3	70	18.48	1489	18.36	1.06	0.90	1.25	0.494
MIR_28_3P	69	17.41	1400	17.55	0.96	0.84	1.09	0.536
MIR_122_5P	72	18.03	1528	17.92	1.05	0.90	1.23	0.545
MIR_128_3P	70	19.04	1392	19.13	0.95	0.80	1.13	0.571
MIR_324_3P	36	18.78	716	18.64	1.06	0.86	1.31	0.583
MIR_1260A	73	18.54	1487	18.46	1.05	0.88	1.26	0.586
LET_7I_5P	57	19.67	1227	19.58	1.05	0.86	1.29	0.623
MIR_582_5P	18	19.89	465	19.71	1.07	0.80	1.44	0.651

	PREVALENT AF CASES		NON-AF CASES					
MICRORNA	N	EXPRESSION MEAN C_Q	N	EXPRESSION MEAN C_Q	OR	95% CI		P-VALUE
MIR_22_3P	73	16.60	1527	16.55	1.03	0.86	1.23	0.778
MIR_424_5P	58	19.26	1241	19.21	1.03	0.84	1.26	0.779
MIR_363_3P	59	19.41	1220	19.45	0.97	0.78	1.21	0.791
MIR_423_5P	65	19.36	1425	19.33	1.02	0.83	1.27	0.829
MIR_494_3P	61	16.86	1155	16.90	0.99	0.89	1.12	0.907
MIR_664B_3	60	16.20	1158	16.24	1.00	0.93	1.07	0.928
MIR_1246	39	18.21	905	18.19	1.00	0.86	1.17	0.956
MIR_32_5P	41	19.98	885	19.98	1.00	0.79	1.28	0.979

* Expression levels are reported in C_q (inversely related to plasma levels). Note that hazard ratio describes the risk in prevalent AF with 1 C_q increase

	INCIDENT AF		NO INCIDENT AF					
**MICRORNA**	N	Expression Mean C_Q	N	Expression Mean C_Q	Hazard Ratio	95% CI		P-Value
**MIR_122_5P**	141	18.12	1387	17.90	1.12	1.00	1.24	0.053
**MIR_93_5P**	137	18.05	1380	17.86	1.14	1.00	1.31	0.058
**MIR_26B_5P**	142	17.62	1403	17.44	1.13	0.99	1.29	0.063
**MIR_30A_5P**	145	17.09	1409	16.90	1.13	0.99	1.29	0.067
**MIR_652_3P**	108	18.32	1108	18.71	0.92	0.84	1.01	0.071
**MIR_150_5P**	141	17.43	1392	17.26	1.10	0.99	1.23	0.089
**MIR_23A_3P**	143	16.06	1405	15.92	1.12	0.97	1.29	0.112
**MIR_30E_5P**	143	17.17	1403	17.02	1.12	0.97	1.29	0.115
**MIR_223_3P**	144	14.96	1413	14.81	1.08	0.98	1.20	0.133
**MIR_17_5P**	138	17.96	1380	17.82	1.11	0.97	1.26	0.144
**MIR_451A**	143	12.18	1406	11.99	1.06	0.98	1.15	0.165
**MIR_19A_3P**	142	15.93	1397	15.79	1.09	0.97	1.22	0.171
**MIR_195_5P**	135	17.04	1373	16.73	1.04	0.98	1.10	0.196
**MIR_148A_3**	137	18.48	1352	18.35	1.08	0.96	1.21	0.201
**MIR_21_5P**	142	15.96	1409	15.83	1.08	0.96	1.22	0.204
**MIR_92A_3P**	143	15.48	1408	15.36	1.09	0.95	1.25	0.206
**MIR_29C_3P**	142	17.03	1388	16.90	1.09	0.95	1.25	0.206
**MIR_191_5P**	141	16.13	1404	15.89	1.06	0.97	1.16	0.219
**MIR_22_3P**	141	16.67	1386	16.54	1.08	0.95	1.22	0.224
**MIR_125A_5**	120	19.50	1227	19.41	1.10	0.94	1.30	0.243
**MIR_28_3P**	129	17.72	1271	17.54	1.06	0.96	1.16	0.247
**MIR_186_5P**	131	19.54	1265	19.43	1.09	0.94	1.28	0.257
**MIR_29A_3P**	140	17.00	1390	16.89	1.08	0.94	1.24	0.264
**MIR_148B_3**	134	18.68	1345	18.58	1.08	0.95	1.22	0.272
**MIR_25_3P**	141	17.08	1409	16.97	1.07	0.95	1.21	0.275
**MIR_27A_3P**	140	17.66	1374	17.55	1.06	0.96	1.17	0.281
**MIR_15B_5P**	142	17.08	1402	16.99	1.08	0.94	1.25	0.284

	INCIDENT AF		NO INCIDENT AF					
MICRORNA	N	Expression Mean C_Q	N	Expression Mean C_Q	Hazard Ratio	95% CI		P-Value
**MIR_3613_3**	70	18.81	710	18.64	1.08	0.94	1.24	0.299
**LET_7G_5P**	114	19.59	1157	19.49	1.08	0.93	1.26	0.337
**MIR_1260A**	137	18.53	1350	18.45	1.07	0.94	1.22	0.338
**MIR_532_3P**	93	19.82	979	19.70	1.07	0.93	1.23	0.353
**MIR_128_3P**	130	19.03	1262	19.14	0.94	0.83	1.07	0.358
**MIR_185_5P**	126	19.12	1289	19.03	1.06	0.93	1.22	0.385
**LET_7A_5P**	122	19.55	1233	19.49	1.07	0.92	1.24	0.390
**MIR_30B_5P**	124	19.77	1231	19.70	1.08	0.91	1.27	0.395
**MIR_323B_3**	86	17.97	877	17.85	1.05	0.94	1.17	0.397
**MIR_126_5P**	144	16.85	1431	16.79	1.06	0.92	1.22	0.443
**MIR_27B_3P**	115	19.06	1218	19.18	0.95	0.82	1.09	0.447
**LET_7D_5P**	121	19.61	1171	19.55	1.06	0.91	1.24	0.450
**MIR_421**	135	16.37	1388	16.21	1.02	0.97	1.07	0.454
**MIR_23B_3P**	127	19.46	1300	19.41	1.06	0.91	1.25	0.464
**MIR_320B**	103	17.08	1047	16.97	1.02	0.96	1.09	0.467
**MIR_194_5P**	131	18.51	1292	18.38	1.04	0.93	1.17	0.474
**MIR_146A_5**	140	18.50	1379	18.44	1.05	0.91	1.22	0.495
**LET_7I_5P**	111	19.63	1116	19.57	1.05	0.91	1.21	0.530
**LET_7B_5P**	118	19.52	1205	19.48	1.05	0.90	1.22	0.550
**MIR_30C_5P**	115	19.84	1202	19.79	1.05	0.88	1.25	0.590
**MIR_140_3P**	129	19.24	1325	19.21	1.04	0.89	1.21	0.646
**MIR_423_5P**	131	19.36	1294	19.33	1.03	0.89	1.20	0.653
**MIR_32_5P**	80	20.03	805	19.97	1.04	0.87	1.23	0.690
**MIR_1246**	74	18.00	831	18.20	0.98	0.88	1.09	0.691
**MIR_126_3P**	143	16.15	1414	16.12	1.03	0.90	1.17	0.713
**MIR_1301_3**	51	20.22	517	20.27	0.95	0.73	1.25	0.731
**MIR_106B_3**	111	17.88	1082	17.96	0.98	0.89	1.09	0.740

	INCIDENT AF		NO INCIDENT AF					
**MICRORNA**	N	Expression Mean C_Q	N	Expression Mean C_Q	Hazard Ratio	95% CI		P-Value
**MIR_628_3P**	65	18.57	676	18.61	0.96	0.77	1.21	0.754
**MIR_382_5P**	120	18.58	1248	18.67	0.98	0.89	1.09	0.756
**MIR_29B_3P**	107	19.61	1172	19.65	0.98	0.82	1.15	0.768
**MIR_664B_3**	103	16.36	1055	16.23	1.01	0.95	1.06	0.792
**MIR_142_5P**	131	18.76	1329	18.75	1.02	0.89	1.16	0.800
**MIR_301B_3**	85	18.20	892	18.22	0.99	0.88	1.11	0.827
**MIR_199A_3**	134	18.63	1355	18.67	0.99	0.86	1.13	0.845
**MIR_424_5P**	110	19.19	1131	19.21	1.01	0.88	1.17	0.872
**MIR_425_5P**	104	19.56	1053	19.57	1.01	0.86	1.19	0.906
**MIR_582_5P**	42	19.67	423	19.72	0.99	0.82	1.19	0.911
**MIR_494_3P**	103	16.94	1052	16.89	1.00	0.92	1.09	0.974
**MIR_144_3P**	106	19.02	1086	19.05	1.00	0.89	1.13	0.985
**MIR_130A_3**	120	19.54	1249	19.55	1.00	0.86	1.17	0.992

* Expression levels are reported in C_q (inversely related to plasma levels). Note that hazard ratio describes the risk in incident AF with 1 C_q increase
