# Supplementary material for: Relations between plasma microRNAs, echocardiographic markers of atrial remodeling, and atrial fibrillation: Data from the Framingham Offspring study
Source: PLoS One. 2020 Aug 19;15(8):e0236960. doi: 10.1371/journal.pone.0236960 (PMC7437902; doi:10.1371/journal.pone.0236960)
Supplement: S1 File — (DOCX) [file pone.0236960.s008.docx]

**Supplement 2: Literature review of mechanisms of microRNA-mediated Atrial Fibrillation**

***MicroRNAs May Mediate Atrial Fibrillation Via Several Mechanisms***

In our study, 6 plasma microRNAs remained significantly associated with LAFI and incident AF after adjustment for covariates and correction for multiple testing.

MicroRNA-20-5p was associated with impaired LAFI and higher risk for both incident and prevalent AF. MicroRNA-20-5p plays a critical role in myocyte differentiation [1]. MicroRNA-20 regulates SMO (Smoothened), a member of the Hh signaling pathway family, key promoters of cardiac myocyte development. In cardiomyocyte differentiated P19 cells, up-regulation of microRNA-20 inhibits cellular proliferation and enhances apoptosis. Furthermore, we previously observed in a separate cohort that plasma levels of microRNA-20 were lower among patients with AF as compared with controls free from this arrhythmia and that microRNA-20 levels changed after catheter ablation [2]. Taken in sum, our findings point to micro-RNA-20-5p as a key marker of structural remodeling and AF vulnerability, perhaps because of microRNA-20’s role as a regulator of atrial myocyte apoptosis.

MicroRNA-26a-5p was also associated with impaired LAFI and higher risk for both incident and prevalent AF. Atrial microRNA-26a-5p expression is lower in canine models of AF [3]. MicroRNA- 26a-5p strongly regulates expression of the *KCNJ2* gene [encoding the inward-rectifier K+ channel responsible for IK1], an ion channel strongly associated with AF-induced electrical remodeling, shortening of action potential duration and promotion of AF [3]. Similarly, Cyclin D2 (*CCND2*), another target of microRNA-26a-5p, is dysregulated with exposure to excessive electrical stimuli [4]. This suggests that gene targets of microRNA-26a-5p may be involved in electrical remodeling or vulnerability to enhanced pulmonary vein automaticity. We previously observed microRNA-26a-5p expression to be lower in the plasma of individuals with AF as compared with hospitalized controls [2]. Lower plasma expression of microRNA-26a-5p in individuals with AF may be caused by or reflect excessive electrical stimulation (i.e. from initial episodes of paroxysmal AF) and drive both electrical (through myocyte KCNJ2) and/or structural (through fibroblast CCND2) remodeling.

MicroRNA-106b-5p was associated with impaired LAFI as well as higher risk for incident and prevalent AF. MicroRNA-106b deficient mice are vulnerable to AF [5]. *Wang et al.* reported that mice with microRNA-106b haploinsufficiency develop sick sinus syndrome and type 2 second- degree atrioventricular block [5]. Heterozygosity of microRNA-106b is associated with electrocardiographic evidence of sinoatrial dysfunction through direct repression of *Shox2* and *Tbx3*, genes involved in sinoatrial node regulation. Further evidence for the role of *Shox2* comes from *Ye et al.,* which revealed that explanted *Shox2* cells from pulmonary vein myocardium had the ability to pace surrounding *Shox2* negative cells and exhibited sinoatrial node-like properties [6]. Lower expression of microRNA-106, as was seen among participants with AF in our study and our prior work, may reflect higher cardiac *Shox2* expression, altered atrial electrophysiological function, and enhanced vulnerability to AF.

Plasma levels of two additional microRNAs, microRNAs 363-5p and 484, were associated with LAFI and incident, but not prevalent AF. We did not examine expression of these microRNAs in our prior study but microRNA-363-3p regulates genes relevant to atrial remodeling, including genes involved in apoptosis (*DNAJB9*), encoding ion channels (*SLC12A5*), and genes responsible for proteosomal degradation (*FBXW7*) [7]. *Wang et al.* has shown that suppression of microRNA-484 may cardiomyocyte apoptosis [8]. However, evidence is scant for the roles of both microRNAs in cardiac development and vulnerability to atrial remodeling. Further research is needed to explore the clinical relevance of these potentially novel AF plasma markers.

**S2 References**

1. Ai F, Zhang Y, Peng B. miR-20a regulates proliferation, differentiation and apoptosis in P19 cell model of cardiac differentiation by targeting Smoothened. Biol Open. 2016 Sep 15;5(9):1260–5.
2. McManus DD, Tanriverdi K, Lin H, Esa N, Kinno M, Mandapati D, et al. Plasma microRNAs are associated with atrial fibrillation and change after catheter ablation (the miRhythm study). Heart Rhythm. 2015 Jan;12(1):3–10.
3. Luo X, Pan Z, Shan H, Xiao J, Sun X, Wang N, et al. MicroRNA-26 governs profibrillatory inward-rectifier potassium current changes in atrial fibrillation. J Clin Invest. 2013 May;123(5):1939–51.
4. Liu Y, Liu W-B, Liu K-J, Ao L, Cao J, Zhong JL, et al. Overexpression of miR-26b-5p regulates the cell cycle by targeting CCND2 in GC-2 cells under exposure to extremely low frequency electromagnetic fields. Cell Cycle Georget Tex. 2016;15(3):357–67.
5. Wang J, Bai Y, Li N, Ye W, Zhang M, Greene SB, et al. Pitx2-microRNA pathway that delimits sinoatrial node development and inhibits predisposition to atrial fibrillation. Proc Natl Acad Sci U S A. 2014 Jun 24;111(25):9181–6.
6. Ye W, Wang J, Song Y, Yu D, Sun C, Liu C, et al. A common Shox2-Nkx2-5 antagonistic mechanism primes the pacemaker cell fate in the pulmonary vein myocardium and sinoatrial node. Dev Camb Engl. 2015 Jul 15;142(14):2521–32.
7. Wong N, Wang X. miRDB: an online resource for microRNA target prediction and functional annotations. Nucleic Acids Res. 2015 Jan;43(Database issue):D146-152.
8. Wang K, Long B, Jiao J-Q, Wang J-X, Liu J-P, Li Q, et al. miR-484 regulates mitochondrial network through targeting Fis1. Nat Commun. 2012 Apr 17;3:781
